# Prevalence of stroke and associated risk factors among middle-aged and older farmers in western China

**DOI:** 10.1186/s12199-017-0621-z

**Published:** 2017-03-15

**Authors:** Song Zhang, Zheng Liu, Yong-Liang Liu, Yu-Ling Wang, Tao Liu, Xiang-Bin Cui

**Affiliations:** 1 Department of Medical Education, 3201 Hospital, Xi’an Jiaotong University Health Science Center, 783 Tianhan Ave, Hanzhong, 723000 Shaanxi China; 2 Department of Pathology and Molecular Medicine, 3201 Hospital, Xi’an Jiaotong University Health Science Center, 783 Tianhan Ave, Hanzhong, 723000 Shaanxi China

**Keywords:** Stroke, Risk factors, Chinese middle-aged and older farmers, Western China, Family history

## Abstract

**Objectives:**

China has the world’s largest population and the stroke has become the leading cause of death in recent years. The purpose of this study was to explore the associations between hypertension, family history of stroke, diabetes mellitus, obesity and stroke among middle-aged and older farmers of western China. A population-based study was conducted from June 2014 to April 2015 in Shaanxi and Sichuan provinces.

**Methods:**

Twenty thousand five hundred twenty-five Chinese middle-aged and older farmers (≥40 years) were recruited to the Qinling-Daba Mountains Region Stroke Study. A structured-questionnaire was used to collect data through face-to-face interviews. Demographic characteristics, risk factors, medical history, and other clinical characteristics were recorded for all participants. The association between hypertension, family history of stroke, diabetes mellitus, obesity and stroke were analyzed by using univariate and multivariate logistic regression analysis.

**Results:**

The stoke prevalence rate was 1380/100,000 in middle-aged and older farmers of western China. The difference in hypertension, diabetes mellitus, obesity and family history between different age groups had statistical significance (*p* < 0.05). The prevalence rate of hypertension and family history of stroke were higher in male population than in the female population. The difference was statistically significant (*p* < 0.05). Univariate logistic regression analysis demonstrated age, gender, hypertension, obesity and family history of stroke were stroke risk factors (*p* < 0.05). Multivariate logistic regression analysis revealed that the odds ratios of family history of stroke, obesity and hypertension were 7.177, 4.389 and 3.647 respectively.

**Conclusions:**

Family history is the strongest stroke risk factor in middle-aged and older farmers of western China.

## Introduction

In China, the stroke mortality rate is approximately 1.6 million annually, approximately 157 cases per 100,000. It has exceeded heart disease to become the leading cause of death [1]. There is also a geographical difference of stroke prevalence in China. Previous studies showed that the stroke prevalence rate was 719 cases per 100,000 population in six China’s cities [2]. Beijing had the highest prevalence rate (1285 cases per 100,000 population per year) [3]. Interestingly, the prevalence rate was low by 95 cases per 100,000 population in Guangxi province (southern China) in comparison to 1249–1285 cases per 100,000 population in Harbin and Beijing cities (northern China) [3, 4]. These studies revealed the fact that the stroke prevalence rate varied widely among different regions within China. In the current study, we for the first time focused on the Chinese middle-aged and older farmers in Shaanxi and Sichuan provinces. These two provinces are located in the western China.

There are approximately 284 million people in western China, including 37 million in Shaanxi province and 80 million in Sichuan province. According to the government database of 2016, over 50% of the population was registered as farmers. However, many of them, especially the young people, are not engaged in agriculture after 1990 due to the urban migration. The Chinese farmers are a very special population who were nearly geographically and socially immobilized in history. Today, in the rural areas of China, most of middle-aged and older farmers are still grouped in the village, engaged in agriculture, and raising living organisms for food. In the past 30 years, the diet and lifestyle of Chinese farmers have changed remarkably as a result of China’s fast economic and social development. The major stroke risk factors, therefore increased substantially, including hypertension, obesity and diabetes mellitus. Over time, there has been a progressive increase in the prevalence of stroke due to the influence of westernized lifestyle, change in eating behavior and increase in the number of older people [5, 6]. Our purpose was to identify the stroke risk factors in middle-aged and older farmers of western China. This stroke epidemiological features can help us identify groups of individuals or regions at higher risk of stroke and therefore push the direction of stroke prevention.

## Materials and methods

### Study area and population

This cross-sectional study was conducted between June 2014 and April 2015. The selection of participants for the study was based on the database in the National Health and Family Planning Commission. The Health Department of Shaanxi and Sichuan provinces contacted the farmers and sent the relevant information to them. The farmers who would like to attend this study have been recruited to the local hospital for participating the study. The participants have been informed about the research and consented to participate. By the way, a physical medical examination and flu vaccine have been freely provided to these participants. A total of 24,147 Chinese farmers (from 40 to 90 year-olds) from Shaanxi and Sichuan provinces were randomly selected for the Qinling-Daba Mountains Region Stroke Study, part of National Stroke Prevention and Screening Project. 20,525 farmers participated (85%). The study population was divided into 5 age groups: 40–49, 50–59, 60–69, 70–79 and 80–89 years. Shaanxi and Sichuan provinces are located in western China with 118.4 million people.

### Case ascertainment

The epidemiologic study was conducted to evaluate stroke and associated risk factors among adults aged over 40 years. The selection of participants was based on the following criteria: (1) their family has lived in the village for at least one hundred years; (2) the people in the village were engaged in agricultural work; (3) the participants have lived in the village for over 20 years in the past 30 years.

### Data collection

The data collection team was made up of two research officers and four trained nurses experienced in community data collection. Each participant underwent a physical examination and was administered a standardized questionnaire by a trained interviewer. The questionnaire included data on age, sex, history of stroke, hypertension, diabetes, and family history of stroke. Informed consent was obtained from all individual participants included in the study. All procedures performed in this study involving human participants was in accordance with the ethical standards of the Xi’an Jiaotong University committee and the Helsinki Declaration of 1975, as revised in 2000. Blood pressures (BP) were measured three times at the right brachial artery using standard mercury Sphygmomanometers after the participant had rested for 10 min in a seated position. Hypertension was defined as systolic BP (average of 3 readings) >140 mm Hg or diastolic BP >90 mm Hg or the use of antihypertensive drugs. Body mass index (BMI) was calculated as body mass divided by the square of the body height (kg/m^2^). Obesity was defined as BMI ≥30 kg/m^2^. Diabetes mellitus was defined as a requirement for diabetes medication or a repeated fasting plasma glucose level exceeding 7.0 mmol/L or 11.1 mmol/L 2 h after eating. Stroke was diagnosed by a neurologist in a hospital at or above the county level based on the self-reported history of stroke and cranial computed tomography or magnetic resonance imaging. Nonfatal ischemic stroke, hemorrhagic stroke, and transient ischemic attack were all included. The family history was defined as a history of stroke in parents, grandparents, aunts or uncles, and first cousins. The smoking and eating habits of these farmers have been observed.

### Statistical analysis

Data were entered into a database using EpiData 3.0 software (EpiData Association Odense, Denmark). All statistical analysis was performed using SPSS (version 18.0; SPSS, Chicago, IL). Data were presented as percentages for categorical variables. Univariate logistic regression analyses were used to determine whether there were differences among the groups. The factors showing statistically significant association with stroke in the univariate analysis were considered for the multivariate analysis. Multivariate logistic regression was performed to assess associations between stroke (dependent variable) and hypertension, family history of stroke, age, gender and obesity. Odds ratio (OR) together with its 95% confidence interval (CI) and *p* values of the final model was presented. *P* less than 0.05 was considered significant, and probability values were two-sided. The age-standardized prevalence rates were calculated by the direct method, using the world population as a standard [7].

## Results

There were 8050 males and 12,475 females with mean age of 59.06 (40–90) years. Females accounted for 60.72% of the population (Table 1). The overall age-adjusted stroke prevalence rate among the Chinese middle-aged and older farmers aged 40 to 90 years was 1380/100,000 corresponding to 118.4 million people in Shaanxi and Sichun provinces. The highest prevalence rates in the present study were found in 70–79 age group (2860/100,000) and the lowest in 40–49 age group (480/100,000). Age-adjusted prevalence rates of stroke among men were higher as rates recorded for women (1870/100,000 compared with 1060/100,000). The age distribution of the number of people with stroke was illustrated in Table 2.

**Table 1:** Demographic characteristics of the study population

Characteristics	Cases, *n*	(%)
Gender		
Male	8062	39.27
Female	12463	60.72
Marital status		
Unmarried	273	1.33
Married	20219	98.51
Widowed	32	0.20
Divorce	1	0.10
Education level		
Primary and less	20388	99.33
Junior high school	125	0.61
High school/college	12	0.10

*n* number

**Table 2:** Age-specific stroke prevalence rates (per 100,000 population per year)

Age (*y*)	Total Number			Patients		Prevalence
	Male, *n*	Female, *n*	Total, *n*	Male, *n* (*PR*)	Female, *n* (*PR*)	Total, *n* (*PR*)
40–49	1681	3302	4983	12(710)	12(360)	24(480)
50–59	2416	3942	6358	38(1570)	40(1010)	78(1230)
60–69	2426	3275	5701	48(1980)	40(1220)	88(1540)
70–79	1290	1546	2836	47(3640)	34(2200)	81(2860)
80–90	249	398	647	6(2460)	6(1570)	12(1920)
Total	8062	12463	20525	151(1870)	132(1060)	283(1380)

*y* years, *n* number, *PR* prevalence rates

The difference in hypertension, diabetes mellitus, obesity and family history of stroke among different age groups had statistical significance (Table 3). The difference in hypertension and family history of stroke between male and female was significant (Table 4).

**Table 3:** Stroke risk factors associated with age groups

Factors	Total (%)	40–49, *n*	50–59, *n*	60–69, *n*	70–79, *n*	80–89, *n*	*χ* ^*2*^	*p*
Hypertension	5409(26.35)	758	1544	1807	1062	238	636.220	<0.001
Diabetes	1248(6.08)	175	367	431	236	39	32.921	<0.001
Obesity	3021(14.72)	807	1011	860	301	42	16.761	<0.001
Family history	531(2.59)	153	172	144	55	7	46.227	0.005

*n* number

**Table 4:** Stroke risk factors associated with sex

Factors	Total, *n* (%)	Male, *n* (%)	Female, *n* (%)	*χ* ^*2*^	*p*
Hypertension	5409(26.35)	2207(27.38)	3202(25.69)	7.147	0.008
Diabetes	1248(6.08)	498(6.18)	750(6.02)	0.218	0.654
Obesity	3021(14.72)	1140(14.14)	1881(15.09)	3.537	0.061
Family history	531(2.59)	239(2.96)	292(2.34)	7.505	0.007

*n* number

Tables 5 and 6 presented the results of univariate and multivariate logistic regression analysis. Univariate logistic regression analysis revealed that *p* value of gender, age, hypertension, obesity and family history of stroke was less than 0.05. Multivariate logistic regression analysis showed that family history was the most important stroke risk factor in Chinese middle-aged and older farmers of western China, followed by obesity and hypertension. Family history of stroke had an OR of 7.177 with 95% CI 5.165 to 9.973. Obesity and hypertension had an OR of 4.389 and 3.647 with 95% CI 3.087 to 6.239 and 2.824 to 4.709, respectively. Interestingly, the results shown that the male has a higher risk of stroke than women.

**Table 5:** Risk factors associated with stroke analyzed by univariate logistic regression

Variables	With Stroke Cases, *n* (%)	Without-stroke Cases *n* (%)	*p* value	OR	95% CI
Age (years)					
40–49	25 (0.122)	4967 (24.20)	<0.001	1.51	1.37–1.68
50–59	74 (0.36)	6282 (30.61)			
60–69	92 (0.45)	5601 (27.29)			
70–79	84 (0.41)	2747 (13.38)			
80–89	7 (0.03)	619 (3.02)			
90	0	26 (0.13)			
Gender					
Male	151 (0.74)	7911 (38.54)	<0.001	1.78	1.41–2.26
Females	132 (0.64)	12331 (60.08)			
Hypertension					
Yes	183 (0.89)	5226 (25.46)	<0.001	5.26	4.11–6.72
No	100 (0.49)	15016 (73.16)			
Diabetes mellitus					
Yes	23 (0.11)	1225 (5.97)	0.15	1.37	0.89–2.11
No	260 (1.27)	19017 (92.65)			
Family history					
Yes	59 (0.29)	472 (2.30)	<0.001	11.03	8.17–14.91
No	224 (1.09)	19770 (96.32)			
Obesity					
Yes	60 (0.29)	2961 (14.43)	0.002	1.57	1.18–2.09
No	223 (1.09)	17281 (84.19)			
Education					
Primary and less	283(1.38)	20105(97.95)	1.00	0.00	-
Junior middle school	0	125(0.61)			
High school or college	0	12(0.05)			
Marriage status					
Unmarried	1(0.00)	272(1.33)	0.25	1.07	0.95–1.20
Married	282(1.37)	19937(97.14)			
Widowed	0	32(0.16)			
Divorced	0	1(0.00)			

*n* number

“-” Missing value

**Table 6:** Stroke risk factors analyzed by using multivariate logistic regression

Variables	RC	SE	Wald *χ* ^2^	*p*	OR	95% CI
Ages	0.294	0.058	25.905	0.001	1.342	1.198–1.502
Family history of stroke	1.971	0.168	137.844	0.004	7.177	5.165–9.973
Obesity	1.479	0.179	67.910	<0.001	4.389	3.087–6.239
Hypertension	1.294	0.130	98.384	<0.001	3.647	2.824–4.709
Gender	−0.570	0.124	21.004	<0.001	0.566	0.443–0.722

*RC* regression coefficients, *SE* standard error, *OR* odds ratio, *CI* confidence interval

## Discussion

This study is the first population-based study of the stroke prevalence in Chinese older farmer population in western China. Our results revealed that the stroke prevalence rate in middle-aged and older farmers of western China was 1380/100,000. The prevalence rate for Chinese farmers was lower than that reported recently by Xun Tang (1420/100,000–1690/100,000) from Beijing Cardiovascular Disease Study [8]. This difference might be due to the fact that the participants were recruited from different region. Beijing is located in the northeast China, whereas Shaanxi and Sichuan are located in the western China. The prevalence of stroke was a tendency for the rates to increase gradually from south to north and to decrease progressively from east to west in China. According to these reports, the reason for this related to the difference of diet, alcohol and cigarette consumption, or prevalence of hypertension in different China region [9, 10]. The stroke prevalence was higher in male than in female and the hypertension and family history was consistent with this (*p* < 0.01).

China now faces similar cardiovascular and stroke risk factors as in the western nations: hypertension, obesity, diabetes mellitus, smoking and physical inactivity. Hypertension, obesity, diabetes mellitus and smoking have been considered to be the four leading causes of stroke in the Chinese population [11–14]. Among them, hypertension remains the most important risk factor for all types of strokes with the highest population-attributable risk at 34.6% [4]. China has hundreds years history of smoking tobacco or some kind of plant leaves according to the local customs. To our observation, most of middle-aged and older farmers in these two provinces smoked beedi, hookah or rollies and the contents were various including herbal fruits and tobacco. This smoking habits appears to vary from region to region, and they are not uniform. For example, some farmers like hookah while some like self-made tobacco. Some farmers smoke the dry leaf of the tree. In our search of the literature, there was no report on the comparison of the health hazards of smoking between these traditional tobacco and cigarette. Furthermore, the dietary habits also vary widely among these farmers. It depends on local traditional habits, personal habit and personal economic status. For example, the main foods of most farmers are rice and vegetables. However, the main vegetables are totally different between some counties in Shaanxi and in Sichuan since the distance between the two counties is one thousand kilometers. Therefore, only hypertension, obesity, diabetes and family history of stroke have been selected to analyze. In our study, for the first time we revealed that family history was the strongest stroke risk factor in middle-aged and older farmers of western China. Hypertension and obesity were the stroke risk factors followed by family history. Interestingly, diabetes was not a stroke risk factor in the middle-aged and older farmers of western China. The reason for this might be due to the fact that the older farmer has their special eating habits based on their living conditions. According to our observation, the main foods of the older farmer were low-fat diet. This was consistent with the previous finding that diabetes prevalence was much lower in farmers than urban residents in China [15].

In recent years, some reports have proved that family history was a stroke risk factor in Chinese population [16, 17]. A study conducted in 2012 demonstrated that family history of stroke was associated with the risk of stroke (OR of 2.74; 95% CI, 1.76–4.26) in people ≥20 years of age [18]. In Japan, family history of stroke was associated with arterial stiffness in middle-aged population (35–69 years old) [19]. In another case-control study of 195 patients (30–79 years old), family history has been found that it was the strongest independent risk factor for subarachnoid haemorrhage [20]. The high waist circumference and social isolation also have been considered to be the risk factors of cardiovascular [21, 22]. However, hypertension has been recognized as the most important stroke risk factor in Chinese population (18–74 years old) [23, 24]. The reason for this may be related to the change of health conditions in the Chinese population in recent years due to obesity, unhealthy eating, and physical inactivity [25, 26]. The physiological changes such as overweight and respiratory diseases mask the role of genetic factors in the development of stroke. Another reason for this may be related to the increased rates of detection of stroke. In recent years, with the improvement in health condition, a large number of slight stroke cases has been identified in Chinese rural region [27]. Moreover, our study was for the first time to investigate the relationship between the stroke and middle-aged and older farmers in western China by using a detailed questionnaire survey. Therefore, it is not surprising that many cases with a family history have been identified.

In summary, the present study demonstrates that family history is the strongest stroke risk factor in middle-aged and older farmers of western China. The stroke prevalence rate is lower in western China than northern China, but is much higher than southern China. This information is useful for planning and implementation of prevention programs and represents the first step toward monitoring the effectiveness of public health strategies to reduce the burden of stroke among middle-aged and older farmers in western China.
